# Submentalizing or Mentalizing in a Level 1 Perspective-Taking Task: A Cloak and Goggles Test

**DOI:** 10.1037/xhp0000319

**Published:** 2016-11-28

**Authors:** Jane R. Conway, Danna Lee, Mobin Ojaghi, Caroline Catmur, Geoffrey Bird

**Affiliations:** 1 MRC Social, Genetic, & Developmental Psychiatry Centre, Institute of Psychiatry, Psychology, & Neuroscience, King’s College London; 2 Department of Psychology, Institute of Psychiatry, Psychology, & Neuroscience, King’s College London; 3 Institute of Cognitive Neuroscience, University College London

**Keywords:** domain-general processing, implicit mentalizing, submentalizing, theory of mind, visual perspective taking

## Abstract

It has been proposed that humans possess an automatic system to represent mental states (‘implicit mentalizing’). The existence of an implicit mentalizing system has generated considerable debate however, centered on the ability of various experimental paradigms to demonstrate unambiguously such mentalizing. Evidence for implicit mentalizing has previously been provided by the ‘dot perspective task,’ where participants are slower to verify the number of dots they can see when an avatar can see a different number of dots. However, recent evidence challenged a mentalizing interpretation of this effect by showing it was unaltered when the avatar was replaced with an inanimate arrow stimulus. Here we present an extension of the dot perspective task using an invisibility cloaking device to render the dots invisible on certain trials. This paradigm is capable of providing unambiguous evidence of automatic mentalizing, but no such evidence was found. Two further well-powered experiments used opaque and transparent goggles to manipulate visibility but found no evidence of automatic mentalizing, nor of individual differences in empathy or perspective-taking predicting performance, contradicting previous studies using the same design. The results cast doubt on the existence of an implicit mentalizing system, suggesting that previous effects were due to domain-general processes.

## Public Significance Statement

The ability to represent in one’s own mind what other people see, think, or believe is important for social interactions and relationships. There is wide agreement that this ‘mentalizing’ ability depends on a late developing, slow and effortful system, but much debate on whether humans also possess a fast and automatic mentalizing system. The present studies tested whether participants automatically represented what an onscreen human avatar could see. Objects’ visibility was manipulated by using either a set of telescopes or goggles. One of each set allowed objects to be seen, and the other did not. Participant response times were predicted to be faster when what they saw corresponded to what the avatar saw, and slower when there was a difference. However, this did not occur, providing no evidence for an automatic mentalizing system, suggesting rather that representing others’ mental states is effortful not automatic.

Mentalizing (also known as ‘theory of mind’) refers to the ascription of mental states, such as beliefs and intentions, to oneself and others. Mentalizing plays a crucial role in social interactions, particularly when seeking to predict, understand, or explain another’s behavior. Although the existence of a late-developing, cognitively demanding, ability to represent mental states in human adults and older children is almost universally accepted, there has been considerable debate regarding the existence of an earlier, more automatic and efficient route by which infants and nonhuman animals may represent beliefs, or even ‘belief-like’ states (Apperly & Butterfill, 2009; Butterfill & Apperly, 2013). This debate has largely been methodological in nature, with several authors claiming evidence for an automatic mentalizing system (sometimes described as an ‘implicit mentalizing’ system—Kovács, Téglás, & Endress, 2010; Qureshi, Apperly, & Samson, 2010; Samson, Apperly, Braithwaite, Andrews, & Bodley Scott, 2010; Senju, Southgate, Snape, Leonard, & Csibra, 2011), whereas others have provided alternative explanations for the effects claimed to support the existence of such a system (Cole, Atkinson, Le, & Smith, 2016; Heyes, 2014a; Phillips et al., 2015; Santiesteban, Catmur, Hopkins, Bird, & Heyes, 2014).

In adults, the ‘dot perspective task’ has provided some of the strongest evidence that mentalizing can occur automatically (Qureshi et al., 2010; Samson et al., 2010). Participants are presented with an image of a blue room with red dots on the wall. In the center a human avatar faces toward the right or left wall. Participants are asked to verify whether a given number cue matches the number of red dots they themselves can see on the walls of the room. Importantly, they are instructed to ignore the avatar and respond based on their own visual perspective. On *consistent* trials the number of dots the participant and avatar can see is the same; on *inconsistent* trials the participant and avatar see a different number of dots (because some of the dots are positioned behind the avatar). Despite being told to ignore the avatar, participants respond faster on consistent trials than on inconsistent trials. This ‘consistency effect’ has been interpreted as evidence for automatic mentalizing: that the avatar’s visual perspective (i.e., mental state) is automatically processed in addition to the participant’s own. It is suggested that on inconsistent trials, resolution of the conflict between the participant’s and avatar’s visual perspectives extends response times.

A limitation of the original dot perspective task’s ability to provide evidence of automatic mentalizing is that it did not include a control condition that could test an alternative ‘submentalizing’ hypothesis (Heyes, 2014a). If mentalizing causes the consistency effect, then the effect should not be observed when the central stimulus is not an appropriate target for the attribution of mental states. However, a recent paper (Santiesteban et al., 2014) demonstrated that the same consistency effect is observed when the central stimulus is an arrow rather than an avatar. These data raise the question of whether the automatic process generating the consistency effect involves mentalizing—specifically, representation of what others can see—or a domain-general nonmentalistic process where, for example, the eyes/nose of the avatar and the point of the arrow act as directional cues that automatically orientate participants’ attention to a subset of the dots, slowing responding on inconsistent trials (Catmur, Santiesteban, Conway, Heyes, & Bird, 2016; Santiesteban et al., 2014). A new experimental manipulation is therefore required to find positive evidence of automatic mentalizing in the avatar condition.

Heyes (1998, 2014b, 2015) proposed such a method, known as the ‘goggles test,’ that has provided the strictest test of mentalizing to date. The goggles test is the most refined of a general class of methods to identify mentalizing which make use of an opaque barrier to determine the ability to represent what another perceives. Barrier methods compare behavior in two situations: when in the presence of another agent with full visual access to the environment, and when in the presence of an agent whose view of the environment is blocked by an opaque barrier. In the goggles version of the test, participants first learn a conditional discrimination between two colored goggles, one of which affords seeing and the other not. Participants learn the affordances of the goggles through their own experience with them. A transfer test then follows where the goggles are placed on another individual and participants have to extrapolate from their own experience to infer what can be seen through each pair of goggles.

It could be argued, however, that successful performance on the goggles test does not provide an unequivocal demonstration of mentalizing. In common with other barrier methods, if a participant has repeated experience of opaque barriers, they may learn that when barriers are placed between an object and another individual then that individual does not interact with the object. This experience may allow them to act *as if* they realize that the individual does not see the object, and that therefore the individual does not know it is there, but does not require the participant to represent the other’s mental state (Penn & Povinelli, 2007). One therefore needs to extend the goggles logic so that the participant encounters two situations in which an agent views a scene through transparent barriers, so that past experience suggests that the barrier affords seeing, but where one of the barriers renders a specific object invisible. This situation has been impossible to instantiate until now, but recent advances in the development of ‘cloaking devices’ to render objects invisible (Choi & Howell, 2014) make it possible.

Therefore, in Experiment 1, rather than giving participants experience of transparent and opaque goggles, we used two ‘telescopes’ within a cloaking device. The lenses in both telescopes were transparent, however because of their respective focal lengths it was possible to manipulate an object’s visibility. An object placed at a specific distance from the focal point of the ‘invisible telescope’ was invisible, even though other objects not placed at that point were visible. All objects were visible when viewed through the ‘visible telescope’ because its lens had a different focal length. This cloaking device allowed us to maintain transparency across conditions while manipulating the visibility of a specific object: in this case, the red dots in the dot perspective task. The use of such a novel apparatus within the dot perspective task means that previous experience of how people interact with objects placed behind an opaque barrier cannot explain any modulation of the consistency effect by the visibility of the dots, and allows the inferred mental state of the avatar to be manipulated while holding all other task and stimulus features constant. The use of two telescopes, both with transparent lenses, one of which renders certain objects invisible, allows for precise manipulation of specific mental state content (i.e., what is seen). In addition, and unlike the goggles manipulation, the fact that participants do not have previous experience of transparent materials able to render specific objects invisible means that any nonmentalistic explanation of their potential impact on the consistency effect based on prior learning becomes untenable.

Problems with prior experience of opacity notwithstanding, two recent studies used variants of the goggles test to determine whether automatic mentalizing underpins performance in the avatar condition of the dot perspective task. Cole et al. (2016) inserted either a transparent barrier or an opaque barrier in front of the avatar stimulus. This design provides a test of the automatic mentalizing hypothesis as if the consistency effect is attributable to the representation of the avatar’s visual perspective, then the consistency effect should be modulated by anything that modulates the avatar’s visual perspective (such as the opacity of the barrier). Counter to the hypothesis that automatic mentalizing underlies performance on the standard version of the task, participants demonstrated an equivalent consistency effect for both types of barrier.

Furlanetto, Becchio, Samson, and Apperly (2016) also implemented a variant of the goggles procedure in which they instructed participants as to the properties of two pairs of goggles, one of which was transparent and one opaque, before allowing them to experience the difference themselves. They then administered the standard version of the dot perspective task with the addition of conditions in which the avatar wore either the opaque or transparent goggles. Participants demonstrated a consistency effect when the avatar wore transparent goggles, but not when the avatar wore opaque goggles; results which are consistent with an automatic mentalizing interpretation.

The contrasting results between the Cole and Furlanetto studies can potentially be explained by a crucial methodological difference relating to the judgments participants were required to make during the dot perspective task. It has long been acknowledged that the ‘acid test’ of automatic mentalizing in this task occurs when participants are required to verify whether the number cue matches the number of dots visible from their own perspective only (as used by Cole et al., 2016). Here, any effect of the avatar meets a strict definition of automatic in which even though participants are never required to judge the number of dots visible from the avatar’s perspective, and doing so hinders performance of the instructed task, their performance is nevertheless influenced by the avatar.

Other variants of the dot perspective task have required participants to verify the number cue from both their own and the avatar’s perspective (as used by Furlanetto et al., 2016). The requirement to adopt both perspectives significantly weakens the claim for automaticity, as participants may experience task carry-over effects on own-perspective trials from avatar-perspective trials (Samson et al., 2010, p. 1259; Santiesteban et al., 2014, p. 934; Schurz et al., 2015, p. 387). Such an effect would be automatic in the sense that adoption of the avatar’s perspective on own-perspective trials is task-irrelevant and interferes with performance, but the automaticity would be an artifact of the testing situation rather than a general feature of human cognition. Thus, it is possible that Furlanetto et al. have shown that a carry-over effect of explicit, nonautomatic mentalizing on the avatar-perspective trials modulates the consistency effect on self-perspective trials; however, *what* process is being modulated cannot be determined by their design: it could be either automatic mentalizing or a domain-general process.

Experiments 2 and 3 were designed to investigate this task carry-over explanation of the Furlanetto et al. (2016) result. Experiment 2 repeated the Furlanetto study using their exact design, stimuli, and procedure but with one key variation: participants were asked to respond from their own perspective only and never from the avatar’s perspective. Experiment 3 was a replication of Furlanetto et al. (2016), in which participants responded from both their own and the avatar’s perspective. Comparison of the results of Experiment 2 and Experiment 3 allows a task carry-over effect to be identified if it is present.

In sum, the current experiments utilized two different visibility manipulations embedded in the dot perspective task to determine whether the consistency effect is modulated by the avatar’s inferred mental state. In contrast to the Cole and Furlanetto studies described above, in Experiment 1 participants were not instructed about the properties of the telescopes, instead they discovered their properties through self-discovery only (as per Heyes, 2014b). In addition, in Experiments 1 and 2 only own-perspective trials were used to limit the potential for task carry-over effects to explain the results. Experiment 3 included both self- and other-perspective trials to determine whether the process underpinning the consistency effect is modulated by a task carry-over effect of explicit, nonautomatic, mentalizing.

## Experiment 1

Experiment 1 implemented a variant on the dot perspective task designed such that, should evidence of mentalizing be observed, this evidence could not be explained by submentalizing factors related to domain-general processes or task carry-over effects. This aim was achieved through the use of two clear glass ‘telescopes’ and the addition of an arrow stimulus as used by Santiesteban et al. (2014).

Participants were given real-life experience of the two telescopes, one visible and one invisible, in a blue room with red dots on the wall. Participants could see the red dots through the visible telescope, but not through the invisible telescope. Participants then completed the dot perspective task with the telescopes inserted in front of the avatar and arrow stimuli. If participants represent what the avatar can see, one would expect a consistency effect when the avatar is looking through the visible telescope because on consistent trials there is no conflict between the participant’s and avatar’s perspectives, but on inconsistent trials responding should be slowed due to the conflict in perspectives. However, a consistency effect would not be expected with the invisible telescope because even when the number of dots visible to the participant equals the number of dots in front of the avatar (‘consistent trials’), the avatar cannot see the red dots through the invisible telescope and therefore the participant’s and the avatar’s perspectives are always in conflict. In effect, the use of the invisible telescope means that all trials are inconsistent, and therefore that response times (RTs) on ‘consistent’ and inconsistent trials should be equivalent. As the arrow is not an appropriate target for the attribution of mental states, no consistency effect should be observed with this stimulus, regardless of telescope type.

In contrast, if the consistency effect in the dot perspective task is a result of nonmentalistic domain-general processes, such as the directionality of the stimulus, then one would expect to observe a consistency effect for both the visible and invisible telescope in both the avatar and arrow conditions, providing that the addition of the telescope stimulus does not impact on the relevant cue characteristic (such as the directionality of either stimulus).

### Method

#### Participants

Forty-nine healthy adults volunteered to take part in this experiment in return for a small monetary sum. Data from six participants were excluded from the analysis, 3 because of a technical fault and a further 3 because of being outliers with respect to accuracy (error rate >25%). The remaining 43 participants (37 female) were aged between 17 and 48 (*M* = 25.72, *SD* = 7.57). The data-stopping rule and sample size were determined prior to data collection and were based on previous research. The target sample size was three times (*n* = 48) the size of the original dot perspective task study (Samson et al., 2010; *n* = 16).

#### Stimuli and apparatus

##### Cloaking device

A real-life replica of the blue room from the computer stimuli of the dot perspective task was built. This room measured 275 mm high by 370 mm wide and was situated on an adjustable stand so participants could place their head inside the room while standing. A telescope mount was placed in the center of the room, 150 mm from its back wall. In the center of the back wall there was a porthole of 45 mm diameter where acetates with red dots on them could be placed. The red dots had a diameter of 8 mm, and there were 3 different acetates, with 1, 2, and 3 dots on them respectively.

A white screen was placed above the room’s back wall to occlude the rest of the device from the participant’s view. A 50-mm-diameter achromatic doublet lens of focal length 200 mm was placed behind this screen in line with the porthole and 255.5 mm from the position of the telescope mount. A blue screen, matched in color to the room, was placed 150 mm from this lens. As the red dots were placed on clear acetates, this blue screen acted as a background so the dots appeared as if they were on the back wall of the blue room.

Four telescopes were used, each comprising a 50-mm-diameter achromatic doublet lens attached to a 3-inch aluminum lens tube. There were two pairs of telescopes. In each pair, the invisible telescope had a focal length of 75 mm and the visible telescope had a focal length of 200 mm. To distinguish the telescopes in each pair, they were covered in yellow or green card. Telescope color was counterbalanced across participants.

**Figure 1 fig1:**
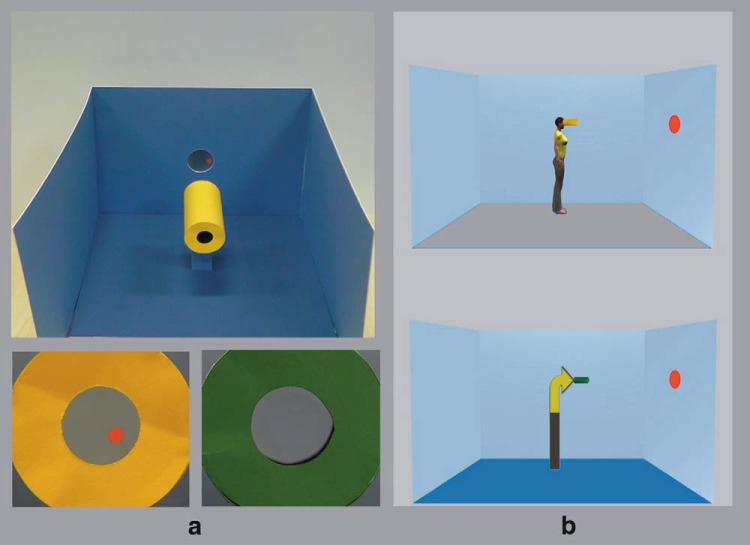
Examples of the cloaking device and computer stimuli in Experiment 1. Panel a (top) shows the blue room apparatus with one red dot present and that the red dot is seen through the visible telescope (panel a bottom left), but not the invisible telescope (panel a bottom right). Sample avatar and arrow stimuli with the telescopes for the computerized dot perspective task are depicted in panel b. See Supplemental Materials (Fig. S.1) and Choi and Howell (2014) for a full explanation of the invisibility effect. See the online article for the color version of this figure.

The set-up of the cloaking device meant that when the visible telescope was placed on its mount in the blue room apparatus, it was possible to see the red dots against the blue background when looking through the telescope; whereas when the invisible telescope was in place, only the blue background was visible when looking through the telescope, the red dots were invisible (see Choi & Howell, 2014; Figure 1; Figure S.1; and Videos S.1 and S.2 in Supplemental Materials for details).[Fig-anchor fig1]

##### Computerized dot perspective task

The computer stimuli were based on those used in Santiesteban et al. (2014; Experiment 2), which were adapted from the original task images used by Samson et al. (2010; Experiment 3). A central stimulus was presented in the middle of a blue room facing either to the right or left. On some trials the stimulus was a human avatar and on others it was an arrow. The avatar and the arrow were matched in height, area, and color. There were two versions of each avatar and arrow: one ‘male’ and one ‘female.’ Participants viewed the central stimulus that matched their own gender. Our stimuli differed from Santiesteban et al. (2014; Experiment 2) in one respect: we inserted the green or yellow telescope into each image type (see Figure 1). On each trial, the green or yellow telescope appeared at the point of the arrow or at the eye of the avatar. Different configurations of red dots appeared on the front and back walls of the blue room. The possible number and configurations of dots were: 1 in front (F) or behind (B); 2F; 1F & 1B; 2B; 3F; 1F & 2B; 2F & 1B; 3B (Santiesteban et al., 2014). Participants completed the task on a laptop computer, and used the ‘K’ key (marked with a ‘1’) to indicate a ‘YES’ response and the ‘L’ key (marked with a ‘2’) to indicate a ‘NO’ response.

#### Procedure

##### Telescope familiarization

Two telescopes of 200-mm focal length, one green and one yellow, were placed on a table in the testing room. The experimenter held up both telescopes and said, “Here are two telescopes, a green telescope and a yellow telescope. Take a look through them.” At this stage, the two telescopes were of the same focal length so the difference in lens strength could not be detected. Participants could look around the testing room at anything they chose. After participants had examined each telescope, the experimenter asked them if they could see through each one, and then instructed them to carry both telescopes to the blue room apparatus which was situated in a separate cubicle within the testing room. Participants were asked to choose which telescope they would like to look through first. The experimenter placed the chosen telescope in the mount. The invisible telescope was covertly swapped for an identical telescope with a focal length of 75 mm (posttest debriefing revealed that no participant was aware of this switch). The experimenter then presented the 3 acetates with red dots to the participant for them to choose which order to view them in. The experimenter placed the first acetate on the back wall of the blue room and, while standing behind the participant, instructed them to look through the telescope. These steps were repeated for each of the 3 acetates for both the visible and the invisible telescope. Then, participants were asked to report what they thought the difference between the two telescopes was. This part of the procedure was video recorded. Following this, participants left the cubicle to complete the computerized task.

##### Dot perspective task

A fixation cross was shown (1250 ms) at the start of each trial, followed by the word ‘YOU’ (1250 ms) to indicate that the participant should judge how many dots they can see from their own perspective; then a number cue between 0 and 3 appeared (750 ms), followed by an image of the blue room. Participants were instructed to press ‘1’ if the number cue matched the number of dots they could see in the image of the blue room, and ‘2’ if the number cue did not match the number of dots they could see in the image. Participants were moved automatically onto the next trial once they made a response or after 2000 ms.

Apart from the inclusion of the telescope stimuli, the experimental design was the same as that used in Santiesteban et al. (2014; Experiment 2) and Samson et al. (2010; Experiment 3), with one further exception: the number of blocks was doubled to achieve the same number of trials per cell of the design as in these previous studies. Thus, participants completed 8 blocks of 52 trials each. Four trials in each block were filler trials in which no dots appeared. On half of the remaining trials, the avatar appeared and on half of these avatar trials the green telescope was present and on the other half the yellow telescope was present. The arrow was present on the remaining trials, half with the green telescope and half with the yellow. Half of the total nonfiller trials were ‘inconsistent’ and the other half ‘consistent’; half required a ‘YES’ response and the other half a ‘NO’ response; and on half the central stimulus faced left and on the other half faced right. Trial types were therefore balanced across blocks. Trial order was pseudorandomized prior to testing to fulfill a rule that a similar trial type should not occur three consecutive times (Samson et al., 2010). Block order was randomized per participant. Participants first completed a practice block of 26 trials with accuracy feedback. No feedback was given on the experimental trials.

##### Manipulation check

Following the 8 experimental blocks, participants completed a further 12 trials in which they were presented with images of the blue room with the avatar stimulus. On half of these trials the yellow telescope was present and on the other half the green telescope was present; half of the trials were inconsistent and the other half were consistent. Different numbers and configurations of red dots were presented on each trial. Participants were asked to respond by pressing the keys 0/1/2/3 to indicate a response to the question: “*How many dots can the woman/man see through the green/yellow telescope*?”

### Results

#### Analysis strategy

In keeping with previous studies, reaction time (RT) data were analyzed from ‘YES’ trials with correct responses only, using a 2 × 2 × 2 repeated measures ANOVA with within-subjects factors of Consistency (Consistent vs. Inconsistent), Stimulus (Avatar vs. Arrow), and Telescope Type (Visible vs. Invisible). The total number of errors was low (*M*_*error rate*_ = 3%) and so accuracy data are reported in the Supplemental Materials; where effects are significant in the error data they are consistent with the RT data, providing no evidence for speed–accuracy trade-offs. Results were also analyzed within a Bayesian framework using JASP (https://jasp-stats.org; JASP Team, 2016), to examine the strength of the evidence in favor of the null and experimental hypotheses. Bayes Factors are particularly relevant to the current analyses as they provide a ratio of the likelihood of the observed data under the null versus alternative hypothesis, whereas *p* values examine the probability of the data given the null hypothesis and therefore cannot discriminate between evidence for the null and no evidence for either the null or alternative hypothesis (Dienes, 2016). Bayes Factors (BF01) are reported below, where values approaching zero indicate that the data provide more evidence in favor of the alternative hypothesis than the null hypothesis, a value of 1 indicates that the null and alternative hypotheses are equally likely given the data, and values above 1 indicate greater support for the null hypothesis. By convention values <1/3 and >3 are taken as evidence in favor of the alternative and null hypotheses, respectively, while values within these boundaries are judged to provide no evidence to favor either the null or alternative hypotheses.

#### Reaction time data

There was a main effect of Consistency, *F*_(1,42)_ = 32.87, *p* < .001, η_ρ_^2^ = .439, BF01 = 4.024 × 10^−14^, whereby RTs (in ms) were significantly faster on consistent trials (*M* = 514, *SE* = 15, *CI* [483, 544]) than on inconsistent trials (*M* = 549, *SE* = 19, *CI* [511, 587]. There was also a main effect of Stimulus, *F*_(1,42)_ = 4.39, *p* = .042, η_ρ_^2^ = .095; RTs were significantly slower on trials on which the avatar was the central stimulus (*M* = 535, *SE* = 17, *CI* [500, 570]) rather than the arrow (*M* = 528, *SE* = 16, *CI* [495, 560]), but this was qualified by the fact that the Bayesian analysis found no support for either the null or alternative hypothesis (BF01 = 1.737). The Consistency x Stimulus interaction was also significant, *F*_(1,42)_ = 6.89, *p* = .012, η_ρ_^2^ = .141, but again the Bayesian analysis indicated no support for either the null or alternative hypothesis (BF01 = 1.182). It should also be noted that after controlling for the overall difference in RT between stimuli, this interaction was no longer significant (*F*_(1,41)_ = 3.18, *p* = .08, η_ρ_^2^ = .072). Crucially, a consistency effect was found for both the avatar stimulus (*F*_(1,42)_ = 31.73, *p* < .001, η_ρ_^2^ = .430, BF01 = 2.411 × 10^−8^) and the arrow stimulus (*F*_(1,42)_ = 21.84, *p* < .001, η_ρ_^2^ = .342, BF01 = 8.056 × 10^−5^). If the inanimate arrow stimulus can produce a consistency effect, then one cannot rely on the simple presence of a consistency effect as evidence for automatic mentalizing, as an arrow cannot ‘see’ the dots and does not have mental states.

**Figure 2 fig2:**
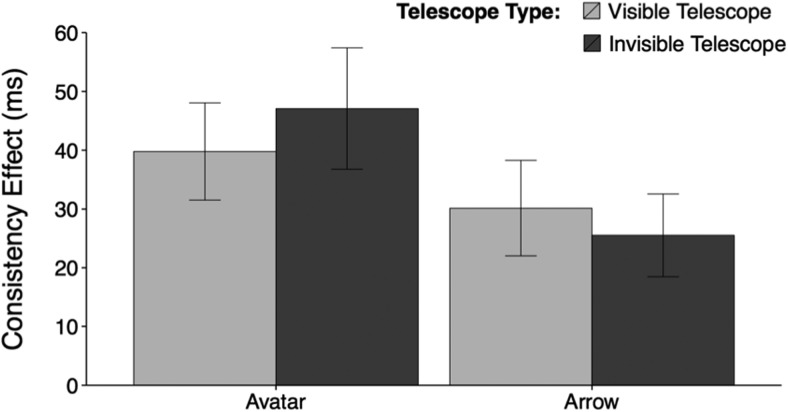
Mean consistency effect for each stimulus and telescope type in Experiment 1. Error bars show the standard error of the mean.

Evidence of mentalizing would be obtained however, if the consistency effect varies as a function of Telescope Type for the avatar but not the arrow. The crucial statistics that would indicate evidence of automatic mentalizing are a significant 3-way interaction between Consistency × Stimulus × Telescope Type, or, less convincingly, a significant Consistency × Telescope Type interaction in the avatar condition only. No such evidence of automatic mentalizing was found however. The consistency effect did not vary as a function of Telescope Type and Stimulus (Consistency × Stimulus × Telescope Type: *F*_(1,42)_ = 0.63, *p* = .43, η_ρ_^2^ = .015, BF01 = 4.293), and in the avatar condition there was no effect of Telescope Type on the consistency effect (Consistency × Telescope Type: *F*_(1,42)_ = 0.48, *p* = .49, η_ρ_^2^ = .011, BF01 = 3.959) (means, standard errors, and 95% confidence intervals for these data are presented in Table S.1. in Supplemental Materials). As can be seen, the Bayes Factors provide support for the null over the alternative hypothesis in each case. Indeed, in the avatar condition, the consistency effect was numerically larger in the invisible telescope condition than in the visible telescope condition—a pattern opposite to that which would be predicted on the basis of automatic mentalizing (see Figure 2).[Fig-anchor fig2]

#### Confirmatory analysis

The logic of the telescope addition to the dot perspective task requires participants to be aware of the nature of each telescope. If participants should forget the fact that one telescope does not allow the red dots to be seen, or forget the mappings between telescope type and color, then it is possible that a Consistency × Stimulus × Telescope Type interaction would not be seen even if participants were automatically mentalizing. Accordingly, a very strict criterion was adopted such that only participants who correctly reported the difference between telescopes at the start of the experiment, and who responded correctly on 12 of 12 of the explicit questions at the end of the procedure (*n* = 21) were included. These participants were explicitly aware of the nature of the telescopes, and which telescope afforded seeing the red dots and which not, at the start and end of the experiment. Even among this highly selected set neither the Consistency × Stimulus × Telescope Type interaction (*F*_(1,20)_ = 0.02, *p* = .88, η_ρ_^2^ = .001, BF01 = 3.272), nor the Consistency × Telescope Type interaction within the avatar condition (*F*_(1,20)_ = 0.02, *p* = .89, η_ρ_^2^ = .001, BF01 = 3.273), was significant.

### Discussion

The introduction of visible and invisible telescopes to the dot perspective taking task allowed a clear prediction to be made: if participants were automatically representing the avatar’s mental state then a consistency effect should have been observed when the avatar was able to see through the visible telescope, but not when the avatar was faced with the invisible telescope, nor when the avatar was replaced with the arrow stimulus, regardless of which telescope accompanied it. Instead, a significant consistency effect was observed in all four conditions. Indeed, the consistency effect was numerically larger when the avatar looked through the invisible telescope than when it looked through the visible telescope, a pattern of data opposite to that predicted by the automatic mentalizing account. Although such a pattern of results is not consistent with the automatic mentalizing hypothesis, it is also inconsistent with the results obtained by Furlanetto et al. (2016). Experiments 2 and 3 investigate a potential explanation for this latter inconsistency.

## Experiment 2

Experiment 1 found no evidence of automatic mentalizing in the dot perspective taking task using a visibility manipulation instantiated using a cloaking device to render the dots invisible. As outlined above, these results are in direct contrast to those obtained by Furlanetto et al. (2016) who used visible and invisible goggles to perform a conceptually similar experiment. We speculated that a possible reason for this discrepancy relates to the participants’ task throughout the experiment. In Experiment 1 participants were required to verify whether the number cue matched the number of dots visible from their perspective only. In contrast, participants in the study of Furlanetto et al. were asked to respond on the basis of both their own and the avatar’s perspective. This feature of the Furlanetto study makes it possible that effects of the avatar’s perspective on own perspective trials were attributable to a task carry-over effect; that as a result of repeated demands to adopt the avatar’s perspective during the task, participants began to do so even on trials where it was not required. Experiment 2 tested for this possibility by implementing the Furlanetto procedure without avatar-perspective trials. Accordingly, participants were given experience of two pairs of goggles, one with transparent lenses through which they could see and the other with opaque lenses through which they could not see. Participants then completed the dot perspective task with the avatar stimulus both without goggles and with opaque and transparent goggles.

If the Furlanetto et al. (2016) effect is truly attributable to automatic mentalizing then one would expect the consistency effect to vary as a function of goggle type. Specifically, automatic mentalizing would be revealed by a consistency effect being observed when the avatar is wearing the transparent goggles and when wearing no goggles, but crucially not when wearing the opaque goggles. In contrast, if the modulation of the consistency effect by goggle type observed on own-perspective trials in the Furlanetto study was attributable to a task carry-over effect, then it should not be evident when participants respond on the basis of their own perspective only. Observation of a consistency effect in all three goggle conditions, including the crucial opaque condition, would indicate that the consistency effect in the dot perspective task is due to nonmentalistic domain-general processes, such as the directionality of the stimulus, and not automatic mentalizing.

Experiment 2 provided a further check on the generalizability of the results of Experiment 1. It could be argued that the cloaking device manipulation used in Experiment 1 is sufficiently novel, or sufficiently outside typical experience, that the automatic mentalizing system cannot represent the way in which it alters visual experience. Although the Confirmatory Analysis reported in Experiment 1 demonstrated that no sign of implicit mentalizing was observed in participants who we could be sure understood the visibility manipulation, the proportion of participants able to meet the strict understanding criterion used in this analysis was surprisingly low. The use in Experiment 2 of transparent and opaque goggles, stimuli with which participants are likely to have much greater experience, should alleviate the concern that the visibility manipulation is outside the realm in which the automatic mentalizing system can operate.

### Method

#### Participants

Sixty-six healthy adults volunteered to take part in this experiment in return for a small monetary sum. Data from nine participants were excluded from the analysis; 4 because they did not follow instructions, and a further 5 for being outliers with respect to accuracy (error rate >25%). The remaining 57 participants (45 female) were aged between 18 and 56 (*M* = 23.37, *SD* = 5.67). The data-stopping rule and sample size were determined prior to data collection and were based on previous research. The target sample size was three times (*n* = 54) the size of the sample in Furlanetto et al. (2016; *n* = 18); see Simonsohn (2015) for discussion of the desirability of a sample at least 2.5 times that of the original study when attempting to replicate an effect.

The same participants completed Experiments 2 and 3. Experiment order was randomly assigned (Experiment 2 first: *n* = 36; Experiment 3 first: *n* = 21). As there were no effects of experiment order, and results for both experiments analyzed separately for each order were consistent with findings from the total sample, these samples were combined and data from the total sample are reported.

#### Stimuli and apparatus

##### Goggles

Four pairs of goggles (two red and two orange) that matched the computerized stimuli from Furlanetto et al.’s (2016) study were used. The external lenses in all goggles were mirrored so that a person’s eyes could not be seen through them. The internal lens in one red and one orange pair of goggles was covered with a blackout material so that they became opaque. The lenses in the other two pairs of goggles were unaltered and therefore remained transparent. The transparent and opaque goggles were indistinguishable when viewed externally.

##### Computerized dot perspective task

**Figure 3 fig3:**
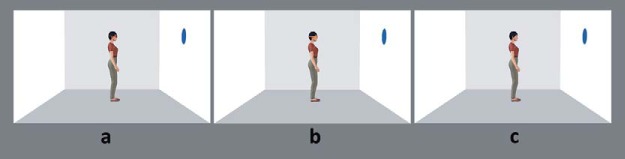
Examples of the computer stimuli in Experiments 2 and 3. Sample avatar stimuli from Furlanetto et al. (2016) with the red (panel a), orange (panel b), and no goggles (panel c) for the computerized dot perspective task in Experiments 2 and 3. See the online article for the color version of this figure.

The computer stimuli were the exact same stimuli as those used in Furlanetto et al. (2016). The Furlanetto task was similar to that outlined in Experiment 1 except that: the room was gray and white with blue dots; the female avatar had a different physical appearance; the fixation cross and word cue were shown for 750 ms each with a 500-ms interstimulus interval; there was no arrow stimulus in this task; and the avatar appeared wearing either red, orange, or no goggles. Goggle type (transparent or opaque) was blocked, whereas whether the avatar was wearing goggles or no goggles was intermixed within a block, as per Furlanetto et al. Sample stimuli are depicted in Figure 3.[Fig-anchor fig3]

#### Procedure

##### Belief induction

Participants were instructed on-screen before the transparent goggle condition, that “In this block the woman/man will sometimes wear orange/red goggles, so she/he will be able to see what is on the wall in front of her/him,” or before the opaque goggle condition, that “so she/he will not be able to see what is on the wall in front of her/him.” Following this, they were instructed “Now you will get first person experience of the visual experience of the woman/man.” The experimenter then gave the participant the goggle type corresponding to the forthcoming condition (opaque or transparent) and asked the participant to look in the direction of the monitor for one minute. There were two separate belief inductions, one for each goggle type prior to the onset of both blocks for that condition (i.e., prior to the start of Block 1 and Block 3).

##### Dot perspective task

The presentation of the dot task was the same as that described in Experiment 1 except for the following changes. As Furlanetto et al. (2016) used 6 blocks of trials in total and had an additional factor that was not included in Experiment 2 (i.e., other-perspective trials on which participants had to respond based on the avatar’s perspective), the current experiment used the self-perspective trials from their study (comprising 3 blocks of trials) and added an additional block to have an equal number for both the opaque and transparent goggles conditions (4 blocks in total).

There were 200 test trials in total, presented in four blocks of 50 trials (2 filler trials per block). Half of the trials in each block were consistent and the other half were inconsistent, half of the trials were matching (i.e., the number cue matched the number of dots participants could see in the image of the room) and the other half mismatching (i.e., the number cue did not match the number of dots participants could see in the image of the room). Within each block 33% of trials showed the avatar stimulus without any goggles and 66% of trials showed the avatar wearing either the red or orange goggles. In contrast to Experiment 1, goggle type (opaque or transparent) was never intermixed within blocks. Block order, opacity order, and goggle color were counterbalanced between participants.

As in Furlanetto et al. (2016), participants first completed 26 practice trials with feedback on trials on which the avatar stimulus had no goggles. No feedback was given on test trials. After the first belief induction phase participants completed the two blocks associated with that goggle condition, then received the second belief induction prior to commencing the final two blocks with the other goggle condition (e.g., two blocks with opaque goggles followed by two blocks with transparent goggles or vice versa). Between each of the four blocks participants were verbally reminded on-screen whether in the upcoming block the woman or man “will/will not be able to see what is on the wall in front of her/him.”

##### Manipulation check

Participants were asked to choose a pair of goggles to wear while performing a visual search task. As in Furlanetto et al. (2016), all participants chose the transparent goggles.

### Results

#### Analysis strategy

As in previous studies, RT data were analyzed from ‘YES’ trials with correct responses only, using a 2 × 3 repeated measures ANOVA with within-subjects factors of Consistency (Consistent vs. Inconsistent) and Goggle Type (No Goggles vs. Transparent Goggles vs. Opaque Goggles). The total number of errors was low (*M*_*error rate*_ = 3.3%) and so accuracy data are reported in the Supplemental Materials; all significant effects in the error data are consistent with the RT data. Where sphericity assumptions were violated, Greenhouse-Geisser corrected values are reported.

#### Reaction time data

There was a main effect of Consistency, *F*_(1,56)_ = 72.81, *p* < .001, η_ρ_^2^ = .565, BF01 = 2.259 × 10^−10^, whereby RTs (in ms) were significantly faster on consistent trials (*M* = 523, *SE* = 11, *CI* [501, 545]) than on inconsistent trials (*M* = 559, *SE* = 13, *CI* [534, 584]). There was no main effect of Goggle Type, *F*_(2,112)_ = 1.90, *p* = .15, η_ρ_^2^ = .033, BF01 = 3.751. The Consistency x Goggle Type interaction was significant, *F*_(2, 112)_ = 4.58, *p* = .012, η_ρ_^2^ = .076, BF01 = 1.378, although the Bayesian analysis provided no support for this effect. The Consistency x Goggle Type interaction was due to a significantly greater consistency effect in the Opaque (*M* = 49, *SE* = 8) than in the Transparent (*M* = 22, *SE* = 6) condition (*Mean*_*diff*_ = 28, *SE* = 9, *p* = .013), whereas no other comparison was significant (Opaque vs. No Goggles: *Mean*_*diff*_ = 14, *SE* = 9, *p* = .424; Transparent vs. No Goggles: *Mean*_*diff*_ = −14, *SE* = 9, *p* = .375).

**Figure 4 fig4:**
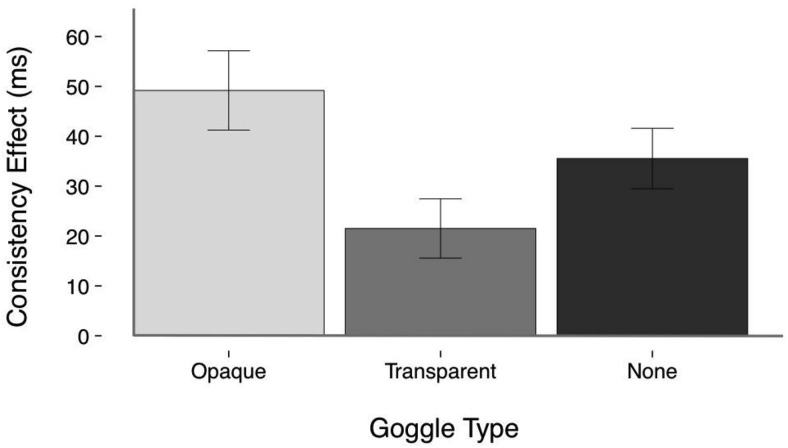
Mean consistency effect for each goggle type in Experiment 2. Error bars show the standard error of the mean.

These data do not support an automatic mentalizing hypothesis, under which a consistency effect would be expected only in the conditions with the transparent goggles and no goggles. Indeed, the significantly greater consistency effect observed when the avatar wore Opaque versus Transparent goggles is directly opposite to what would be expected under the automatic mentalizing account. The lack of support for automatic mentalizing is evidenced by a significant consistency effect in all three conditions: when the avatar was wearing no goggles (*F*_(1,56)_ = 34.36, *p* < .001, η_ρ_^2^ = .380, BF01 = 2.435 × 10^−5^), transparent goggles (*F*_(1,56)_ = 13.12, *p* = .001, η_ρ_^2^ = .190, BF01 = 0.025), and, crucially, opaque goggles (*F*_(1,56)_ = 38.18, *p* < .001, η_ρ_^2^ = .405, BF01 = 8.198 × 10^−6^) (see Figure 4). Means, standard errors, and 95% confidence intervals for these data are presented in Table S.2. in Supplemental Materials.[Fig-anchor fig4]

### Discussion

The results of Experiment 2 provide no support for the hypothesis that automatic mentalizing is responsible for the consistency effect in the dot perspective task. Instead, like the results of Experiment 1 and Cole et al. (2016), they are consistent with a submentalizing perspective in which domain-general processes such as attentional orienting underpin the consistency effect. Furthermore, alleviating any concerns that the cloaking device visibility manipulation in Experiment 1 was too novel or obtuse for the automatic mentalizing system to deal with, results were obtained with familiar materials in a familiar situation, and with explicit instructions as to the properties of the goggles.

## Experiment 3

The results of Experiment 2 are consistent with the hypothesis that the positive evidence of automatic mentalizing reported by Furlanetto et al. (2016) is a task-specific product of a design in which participants are asked to adopt both their own perspective and that of the avatar. To further test this hypothesis, the participants from Experiment 2 also completed Experiment 3, which consisted of a straight replication of the Furlanetto et al. study including both self and avatar perspective trials. Comparison of the results of Experiment 2 and 3 will therefore enable the identification of a task carry-over effect should one exist. Evidence that the submentalizing process underpinning the consistency effect on self-perspective trials can be moderated by a carry-over effect of explicit, nonautomatic mentalizing on avatar-perspective trials would be demonstrated by the observation of a consistency effect in the crucial opaque goggles condition on self-perspective trials in Experiment 3.

Experiments 2 and 3 also investigated individual differences in the size of the consistency effect. A recent paper found that, on self-perspective trials, the consistency effect in the avatar condition was positively correlated with the perspective-taking and empathic concern subscales of the self-report ‘Interpersonal Reactivity Index’ questionnaire (IRI: Davis, 1983), whereas the consistency effect in an arrow, and a rectangular stimulus condition showed no such relationships (Nielsen, Slade, Levy, & Holmes, 2015). Nielsen et al. suggested that these results imply that consistency effects in the avatar condition reflect distinctly social processes that do not operate when consistency effects are observed with nonsocial stimuli. Participants in the current experiments (2 and 3) also completed the IRI to investigate whether the consistency effect in the avatar condition varies according to empathy and perspective-taking.

### Method

The method for Experiment 3 was the same as that for Experiment 2 with the following exceptions described below and was an exact replication of that of Furlanetto et al. (2016), using the same stimuli, design, and procedure.

#### Dot perspective task

There were 300 test trials presented in six blocks of 50 trials (including 2 filler trials per block). There were 3 blocks per goggle condition. There was an additional factor of Perspective with 2 levels, Self and Other. Half of all trials were Self and the other half were Other trials. The word cue ‘YOU’ indicated that the participant should judge how many dots they can see from their own perspective (as in Experiments 1 and 2), the word cue ‘SHE/HE’ indicated that the participant should judge how many dots the avatar can see from the avatar’s perspective. Self and Other trials were intermixed within blocks.

#### Interpersonal reactivity index

On completion of the study, participants were asked to complete the perspective-taking (PT) and empathic concern (EC) subscales of the IRI via an online link to the questionnaire. Each subscale had 7-items scored on a 5-point Likert scale (0 = ‘does not describe me well’; 4 = ‘describes me very well’), and measured the tendency to adopt others’ point of view (PT: α *= .72),* and have concern or compassionate feelings for others (EC: α = .78; Davis, 1983). Sample items included “when I am upset at someone, I usually try to ‘put myself in his shoes’ for a while” (PT), and “I would describe myself as a pretty soft-hearted person” (EC).

### Results

#### Analysis strategy

As in previous studies, RT data were analyzed from ‘YES’ trials with correct responses only, using a 2 × 2 × 3 repeated measures ANOVA with within-subjects factors of Consistency (Consistent vs. Inconsistent), Perspective (Self vs. Other), and Goggle Type (No Goggles vs. Transparent Goggles vs. Opaque Goggles). The total number of errors was low (*M*_*error rate*_ = 6.4%) and so accuracy data are reported in the Supplemental Materials; where effects are significant in the error data they are consistent with the RT data. Where sphericity assumptions were violated, Greenhouse-Geisser corrected values are reported.

The relationship between the consistency effects and the perspective-taking and empathic concern subscales of the IRI were examined as in the study by Nielsen et al. (2015), using one-tailed Pearson’s correlations, and using Bayesian correlations. Analyses were conducted for both overall consistency effects and by goggle type for the self-perspective trials from Experiment 2 and Experiment 3 separately, and the other-perspective trials from Experiment 3.

#### Reaction time data

There was a main effect of Consistency, *F*_(1,56)_ = 75.01, *p* < .001, η_ρ_^2^ = .573, BF01 = 1.029 × 10^−11^, whereby RTs (in ms) were significantly faster on consistent trials (*M* = 602, *SE* = 15, *CI* [573, 632]) than on inconsistent trials (*M* = 660, *SE* = 19, *CI* [622, 697]). There was also a main effect of Perspective, *F*_(1,56)_ = 39.69, *p* < .001, η_ρ_^2^ = .415, BF01 = 2.575 × 10^−9^, with faster responding on Self trials (*M* = 605, *SE* = 16, *CI* [573, 637]) than on Other trials (*M* = 657, *SE* = 18, *CI* [621, 692]). The Consistency × Perspective interaction was significant, *F*_(1,56)_ = 11.22, *p* = .001, η_ρ_^2^ = .167, BF01 = 0.064, with a larger consistency effect in the Other condition (*M* = 80, *SE* = 10, *CI* [61, 99]) than the Self condition (*M* = 35, *SE* = 9, *CI* [16, 53]). There was a significant Consistency × Perspective × Goggle Type interaction, *F*_(1.6, 92.2)_ = 4.86, *p* = .015, η_ρ_^2^ = .080, BF01 = 0.631, which was not supported by the Bayesian analysis.

**Figure 5 fig5:**
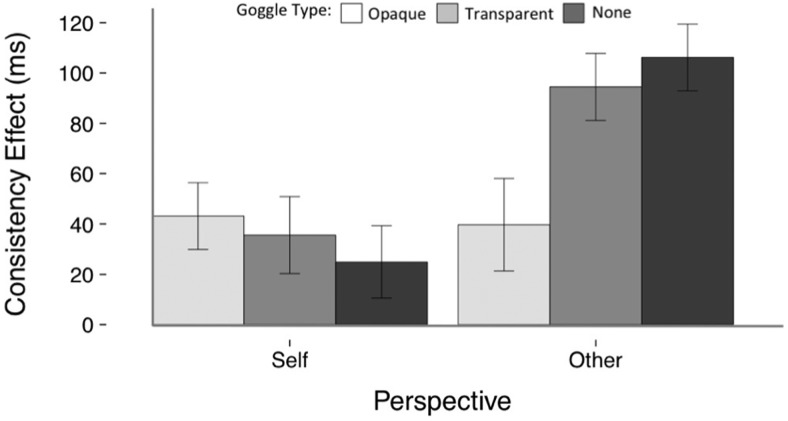
Mean consistency effect for each perspective and goggle type in Experiment 3. Error bars show the standard error of the mean.

The Consistency × Perspective × Goggle Type interaction was driven by a Consistency × Goggle Type interaction that was significant in the Other condition, *F*_(2, 112)_ = 6.042, *p* = .003, η_ρ_^2^ = .097, BF01 = 0.285, driven in turn by the fact that the consistency effect in the Opaque Goggles Other condition was significantly smaller than in the Transparent Goggles Other condition, *F*_(1, 56)_ = 5.74, *p* = .020, η_ρ_^2^ = .093, BF01 = 1.140, and than in the No Goggles Other condition, *F*_(1, 56)_ = 9.18, *p* = .004, η_ρ_^2^ = .141, BF01 = 0.376, although neither of these simple contrasts were supported by the Bayesian analysis (see Figure 5). The difference in the size of the consistency effect between the Transparent Goggles Other and No Goggles Other conditions was not significant, *F*_(1, 56)_ = 0.555, *p* = .459, η_ρ_^2^ = .010, BF01 = 4.241.[Fig-anchor fig5]

The consistency effect observed on such Other perspective trials, that is, when judging the avatar’s perspective, is an example of egocentric intrusion (Samson et al., 2010). On these trials the participant is explicitly instructed to adopt the avatar’s perspective and they are slower to do so when the avatar’s perspective is inconsistent with their own than when it is consistent. Although this effect is interesting, it does not bear upon whether the avatar’s perspective is automatically represented on self-perspective trials. The reduction in the egocentric intrusion effect with opaque goggles is encouraging however, as it suggests that, when explicitly instructed to adopt the avatar’s perspective, participants were representing that the avatar could not see any dots when wearing the opaque goggles. Therefore what were previously ‘consistent’ trials were now in fact inconsistent, as when wearing opaque goggles the avatar never saw any dots on the wall it was facing whereas the participant did on all ‘consistent’ trials that were not filler trials (note that on filler trials no dots appeared; 12/300 trials were fillers and these were not analyzed). Therefore for all of the ‘consistent’ trials analyzed in the opaque goggle condition, the avatar’s and participant’s perspectives were conflicting, thus slowing responding.

In contrast, on Self-Perspective trials the Consistency × Goggle Type interaction was not significant, *F*_(2, 112)_ = 0.48, *p* = .619, η_ρ_^2^ = .009, BF01 = 12.643. The consistency effect did not vary as a function of goggle type, and there was a significant consistency effect in the Opaque Goggles Self condition, *F*_(1, 56)_ = 10.58, *p* = .002, η_ρ_^2^ = .159, BF01 = 0.064, in the Transparent Goggles Self condition, *F*_(1, 56)_ = 5.434, *p* = .023, η_ρ_^2^ = .088, BF01 = 0.505, and a marginally significant effect in the No Goggles Self condition, *F*_(1, 56)_ = 3.00, *p* = .089, η_ρ_^2^ = .051, BF01 = 1.398 (see Figure 5). Note that of the consistency effects in the individual conditions, only that in the Opaque Self condition was supported by the Bayesian analysis, and the Bayesian analysis provided strong support for the lack of any effect of Goggle Type on consistency. Means, standard errors, and 95% confidence intervals for these data are presented in Table S.3. in Supplemental Materials.

The self-perspective trials from Experiment 3 and Experiment 2 (which included only self-perspective trials) were compared to examine whether the Consistency × Goggle Type interaction varied as a function of Experiment. However, the Consistency × Goggle Type × Experiment interaction was not significant, *F*_(2, 112)_ = 0.762, *p* = .469, η_ρ_^2^ = .013, BF01 = 11.923, providing no evidence that task carry-over effects influence the consistency effect.

#### Interpersonal reactivity index

Forty-five participants responded to the questionnaire. In the full data set, there were no significant correlations (all *p*s > .05). In a reduced data set (*n* = 35), from which outliers were removed using the 1.5 interquartile range rule (Tukey, 1977), the only significant correlation observed prior to correcting for multiple testing was a positive relationship between empathic concern and the consistency effect on other-perspective trials in the no goggles condition, *r* = .35, *p* = .02. The interpretation of this correlation is unclear, as in the conceptually similar transparent goggles condition (in which the avatar can also ‘see’) it was not observed, *r* = −.04, *p* = .41. After correcting for multiple comparisons it no longer reached significance. Bayesian analyses showed no support for any correlations in both the full and reduced data set. It is clear, therefore, that these results do not support those observed by Nielsen et al. (2015).

### Discussion

Experiment 3 represented a replication of Furlanetto et al. (2016) with greater power to detect any effects, if present. Despite this, it was not possible to replicate the original results; the magnitude of the consistency effect on self-perspective trials was not modulated as a function of whether the avatar was wearing transparent or opaque goggles. In contrast, strong evidence was obtained from the Bayesian analysis of these data that the consistency effect was *not* modulated by which goggles the avatar was wearing. That the avatar’s visual perspective was manipulated without any effect on participants’ responding on self-perspective trials indicates that automatic representation of the avatar’s mental state does not generate the consistency effect.

The cross-experimental comparison, demonstrating that the consistency effect by goggle type interaction on self-perspective trials is similar in both the self-perspective-only experiment (2) and in the self condition from the mixed-perspective experiment (3), suggests that in Experiment 3, the consistency effect on self-perspective trials was not affected by task carry-over effects from other-perspective trials. Given our larger sample size, our results are therefore more consistent with those from Furlanetto et al. (2016) reflecting a false positive, rather than being attributable to carry-over effects.

In contrast to the findings of Nielsen et al., these data showed no relationship between empathic concern or perspective-taking and the consistency effect. The data therefore do not support the claim that consistency effects for avatar stimuli involve specific mentalistic, or general social, processes.

## General Discussion

The novel invisibility manipulation used in Experiment 1 allowed us to develop an experimental paradigm in which, should evidence consistent with automatic mentalizing have been found, one could reasonably claim that a submentalizing process could not have been responsible for the observed results. In contrast, we found no evidence that participants were automatically representing what the avatar can see in the dot perspective task. Whether the avatar was looking through a telescope through which they either could, or could not, see the red dots made no difference to the size of the consistency effect, a finding which runs counter to any explanation of the consistency effect being due to the representation of what the avatar can see. Similarly, replicating Santiesteban et al. (2014), a consistency effect was also observed for the arrow stimulus. Furthermore, our reexamination of the design and procedure used by Furlanetto et al. (2016) found no support for the claim that ascription of mental states underpins the consistency effect, nor for the possibility that this effect could be modulated by a task carry-over effect of explicit mentalizing (Experiments 2 and 3). Together these findings suggest that domain-general nonmentalistic processes, such as automatic directional cueing, underpin the consistency effect previously found using the dot perspective task.

The current Experiments 2 and 3 also showed no relationship between the size of the consistency effect and individual differences in empathic concern or perspective-taking, and therefore do not support the suggestion by Nielsen et al. (2015) that consistency effects in the avatar condition reflect distinctly social processes. As further support of this claim, Nielsen et al. also pointed to a significantly larger consistency effect on self-perspective trials in the avatar (i.e., social) condition compared with two nonsocial conditions (an arrow and a rectangle). However, the avatar stimulus was significantly larger than the arrow and rectangle stimuli, which were comparable in size, and therefore it is possible that the larger consistency effect in the avatar condition was a result of the size of the central stimulus rather than its social aspects. This confound, and the lack of replication in the current experiments, suggests that processes underlying the consistency effect are not social in nature.

In the automatic (or ‘implicit’) mentalizing literature, a distinction is often made between ‘Level 1’ and ‘Level 2’ perspective taking, where Level 1 refers to the ability to “infer what object another person does and does not see” (Flavell, Abrahams Everett, Croft, & Flavell, 1981, p. 99), and Level 2 refers to knowing “that an object simultaneously visible to both the self and the other person may nonetheless give rise to different visual impressions or experiences in the two if their viewing circumstances differ” (Flavell et al., 1981, p.99). Level 1 perspective taking thus concerns the visibility of an object, while Level 2 perspective taking concerns its appearance. It has been claimed that the automatic and efficient route to belief or belief-like state representation is limited to Level 1 perspective taking only (Apperly & Butterfill, 2009; Butterfill & Apperly, 2013; Qureshi et al., 2010; Surtees, Butterfill, & Apperly, 2012). The dot task is a measure of Level 1 perspective taking as, under the mentalizing account, the consistency effect depends on inferring that the avatar does see the dots on the wall in front of them but does not see the dots on the wall behind them (Apperly & Butterfill, 2009; Furlanetto et al., 2016; Qureshi et al., 2010; Samson et al., 2010; Surtees, Butterfill, & Apperly, 2012).

Crucially, the introduction of a visibility manipulation, as in the current studies and in the studies by Cole et al. (2016) and Furlanetto et al. (2016), does not alter the level of perspective taking of the dot task; rather, it manipulates Level 1 perspective taking: whether another person can see an object seen by oneself. The invisible telescope does not change the appearance of the dots in a way that would qualify for Level 2 perspective taking (e.g., by making them change color while remaining jointly visible to both avatar and participant). The invisible telescope changes the *visibility* of the dots, not their *appearance,* therefore allowing a manipulation of Level 1 perspective taking.

The current experiments add to an emerging literature that reexamines claims of automatic mentalizing as a domain-specific process of mental state representation (Phillips et al., 2015; Santiesteban et al., 2014; Schneider, Lam, Bayliss, & Dux, 2012). A recent reexamination (Phillips et al., 2015) of a different task, first used to support claims of automatic mentalizing in adults and 7-month-old infants (Kovács et al., 2010), demonstrated that the observed effects result from an attention-check rather than automatic mentalizing. The current Experiment 1 goes beyond the analysis of existing effects however, by providing a manipulation by which automatic mentalizing could be detected, if present. Even if it were possible that automatic mentalizing might occur but not interfere with the dot task, the current experiments invalidate the mentalizing interpretation of the consistency effect, showing it is not caused by interference from spontaneous computation of the avatar’s conflicting visual perspective.

The finding that mental states are not necessarily represented in tasks putatively assumed to measure automatic mentalizing has profound implications. Evidence of automatic mentalizing has been used in support of claims including its evolutionary significance as a uniquely human adaptation (Kovács et al., 2010), specific deficits in those with Autism Spectrum Disorder (Senju, Southgate, White, & Frith, 2009), and the presence of a dual-process system for mentalizing (Apperly & Butterfill, 2009; Apperly, 2011). These data suggest that mentalizing may not be as pervasive as previously assumed (Apperly, 2011).

Our findings also contribute to the intriguing possibility that what has been termed ‘automatic mentalizing’ might in fact be entirely accounted for by domain-general processes and, although someone may act *as if* they understand another person’s mental state, no mental states are actually represented (Heyes, 2014a). This opens up new avenues for research to investigate how cultural learning may underpin the development of a full-blown *explicit* mentalizing ability, what ontogenetic experiences enhance or impair this ability, and what factors, such as motivation or intelligence, influence individual differences in the degree of mentalizing skill and the degree to which this skill is applied in everyday life.

## Supplementary Material

10.1037/xhp0000319.supp
