# Supplementary material for: Submentalizing or Mentalizing in a Level 1 Perspective-Taking Task: A Cloak and Goggles Test
Source: J Exp Psychol Hum Percept Perform. 2016 Nov 28;43(3):454–65. doi: 10.1037/xhp0000319 (PMC5327864; doi:10.1037/xhp0000319)
Supplement: Supplementary file 1 [file zfn010163517so4.doc]

**Supplemental Materials**

**Submentalizing or Mentalizing in a Level 1 Perspective-Taking Task: A Cloak and Goggles Test**

**by J. R. Conway et al., 2016, *JEP: Human Perception and Performance***

**http://dx.doi.org/10.1037/xhp0000319**

**Figure S.1.** **Schematic diagram of the cloaking device in Experiment 1**


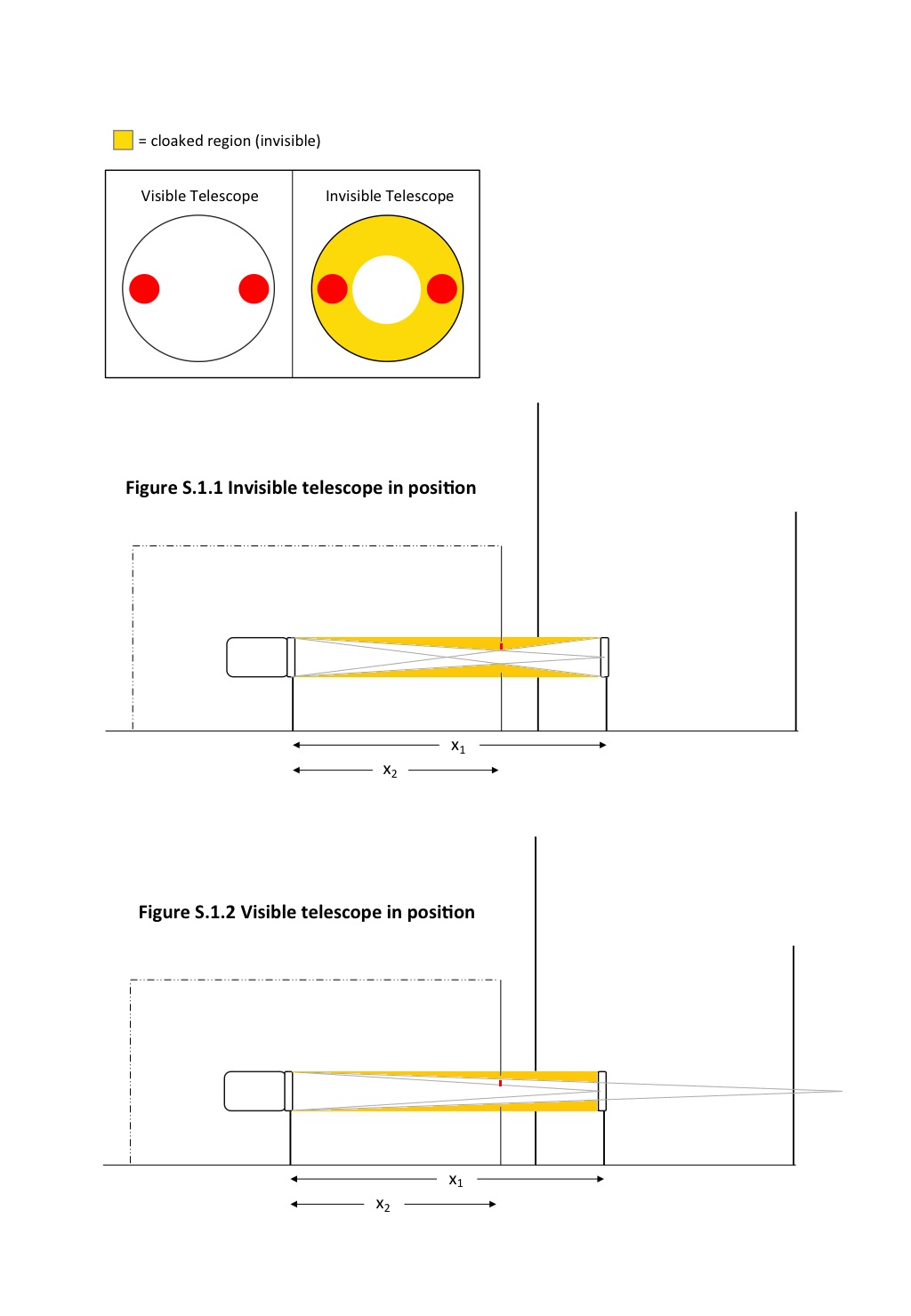


*Figure S.1.* A diagram of the cloaking device. The dashed line represents the outline of the blue room. Participants looked horizontally through the system from the left hand side of the diagram. Distances x_1_ = 255.5mm and x_2_ = 150mm. The telescope was placed on a mount. A high white screen was situated behind the back of the blue room so that the remaining apparatus was occluded from the participant’s view. A 45mm diameter circular hole was cut into this white screen and the back wall of the blue room. Transparent acetates with opaque red dots were placed on the back wall of the blue room so that they appeared within this circle. A blue screen was situated at the end of the system to act as the background when looking through the system. Figure S.1.1 shows the region cloaked by the invisible telescope (75mm focal length). The red dot falls within the cloaked region when viewed through the invisible telescope and therefore cannot be seen (see also Video S.2). Figure S.1.2 shows the region cloaked by the visible telescope (200mm focal length). The red dot does not fall within the cloaked region when viewed through the visible telescope and therefore can be seen (see also Video S.1 and Choi & Howell, 2014 for further details).

**Table S.1.**

*Experiment* *1 Means (M), Standard Errors (SE) and 95% Confidence Intervals (CI) for Reaction Time Data, in milliseconds, for each Trial Type.*

|  | Consistent | | |  | Inconsistent | | |
| --- | --- | --- | --- | --- | --- | --- | --- |
|  | *M* | *SE* | 95% *CI* |  | *M* | *SE* | 95% *CI* |
| Telescope Type | Avatar | | | | | | |
| Visible | 514 | 15 | [483, 545] |  | 554 | 20 | [514, 594] |
| Invisible | 512 | 16 | [481, 544] |  | 560 | 22 | [516, 604] |
|  | Arrow | | | | | | |
| Visible | 512 | 14 | [484, 541] |  | 543 | 19 | [504, 581] |
| Invisible | 515 | 17 | [482, 549] |  | 541 | 17 | [506, 576] |

**Table S.2.**

*Experiment* *2 Means (M), Standard Errors (SE) and 95% Confidence Intervals (CI) for Reaction Time Data, in milliseconds, for each Trial Type.*

|  | Consistent | | |  | Inconsistent | | |
| --- | --- | --- | --- | --- | --- | --- | --- |
|  | *M* | *SE* | 95% *CI* |  | *M* | *SE* | 95% *CI* |
| Goggle Type | Self | | | | | | |
| Opaque | 512 | 10 | [491, 533] |  | 561 | 15 | [532, 591] |
| Transparent | 538 | 13 | [513, 564] |  | 560 | 14 | [532, 587] |
| None | 520 | 12 | [496, 544] |  | 555 | 13 | [530, 581] |

**Table S.3.**

*Experiment* *3 Means (M), Standard Errors (SE) and 95% Confidence Intervals (CI) for Reaction Time Data, in milliseconds, for each Trial Type.*

|  | Consistent | | |  | Inconsistent | | |
| --- | --- | --- | --- | --- | --- | --- | --- |
|  | *M* | *SE* | 95% *CI* |  | *M* | *SE* | 95% *CI* |
| Goggle Type | Self | | | | | | |
| Opaque | 589 | 19 | [551, 626] |  | 632 | 22 | [588, 675] |
| Transparent | 584 | 14 | [556, 612] |  | 620 | 22 | [576, 663] |
| None | 591 | 17 | [558, 624] |  | 616 | 20 | [577, 656] |
|  | Other | | | | | | |
| Opaque | 650 | 26 | [598, 701] |  | 690 | 30 | [630, 749] |
| Transparent | 589 | 15 | [559, 620] |  | 684 | 19 | [647, 721] |
| None | 611 | 15 | [580, 641] |  | 717 | 20 | [677, 757] |

**Accuracy Data**

**Table S.4.**

*Experiment* *1 Accuracy Results from Repeated Measures ANOVA with Factors Consistency, Stimulus, and Telescope Type.*

|  | *F* | *p* | *η_ρ_^2^* | *BF01* |
| --- | --- | --- | --- | --- |
| Consistency | 15.10 | < .001 | .264 | 0.006 |
| Stimulus | 1.60 | .213 | .037 | 4.234 |
| Telescope Type | 0.973 | .329 | .023 | 4.729 |
| Consistency x Stimulus | 0.258 | .614 | .006 | 5.538 |
| Consistency x Telescope Type | 3.540 | .067 | .078 | 1.200 |
| Stimulus x Telescope Type | 0.328 | .570 | .008 | 5.610 |
| Consistency x Stimulus x Telescope Type | 0.006 | .938 | .000 | 4.361 |

*Note.* Consistency x Telescope Type in the Avatar condition: *F* _(1,42)_ = 1.40 *p* = .244, η_ρ_^2^ = .032, BF01 = 2.308; and in the Arrow condition: *F* _(1,42)_ = 2.54, *p* = .119, η_ρ_^2^ = .057, BF01 = 4.152.

**Figure S.2.** ***Experiment* 1 Mean Number of Errors** **for Each Consistency, Stimulus and Telescope Type**


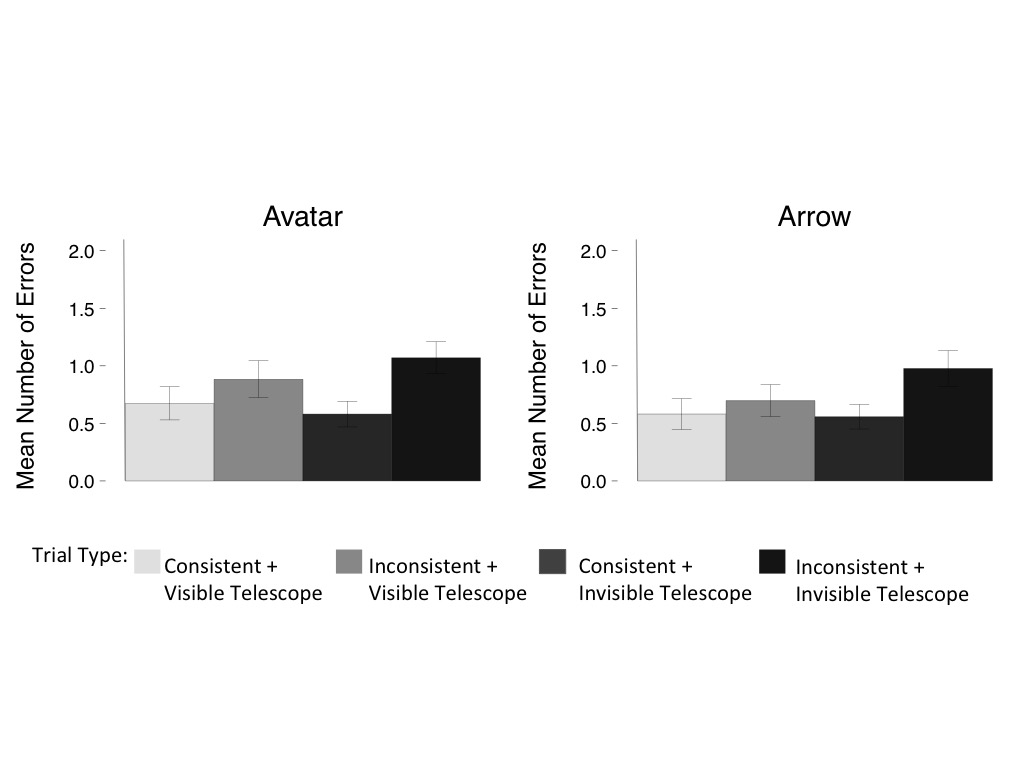


*Figure S.2.* Error bars show the Standard Error of the Mean.

**Table S.5.**

*Experiment* *2 Accuracy Results from Repeated Measures ANOVA with Factors Consistency, and Goggle* *Type.*

|  | *F* | *p* | *η_ρ_^2^* | *BF01* |
| --- | --- | --- | --- | --- |
| Consistency | 10.58 | .002 | .159 | 0.020 |
| Goggle Type | 0.633 | .516 | .011 | 19.485 |
| Consistency x Goggle Type | 3.323 | .040 | .056 | 0.864 |

*Note.* Consistency x Goggle Type for Opaque vs Transparent Goggles: *F* _(1,56)_ = 2.603, *p* = .112, η_ρ_^2^ = .044, BF01 = 1.382; Opaque vs No Goggles: *F* _(1,56)_ = 6.871, *p* = .011, η_ρ_^2^ = .109, BF01 = 2.405; and Transparent vs No Goggles: *F* _(1,56)_ = 0.665, *p* = .418, η_ρ_^2^ = .012, BF01 = 1.973.

**Figure S.3.** ***Experiment* 2 Mean Number of Errors** **for Each Consistency and Goggle Type**

*
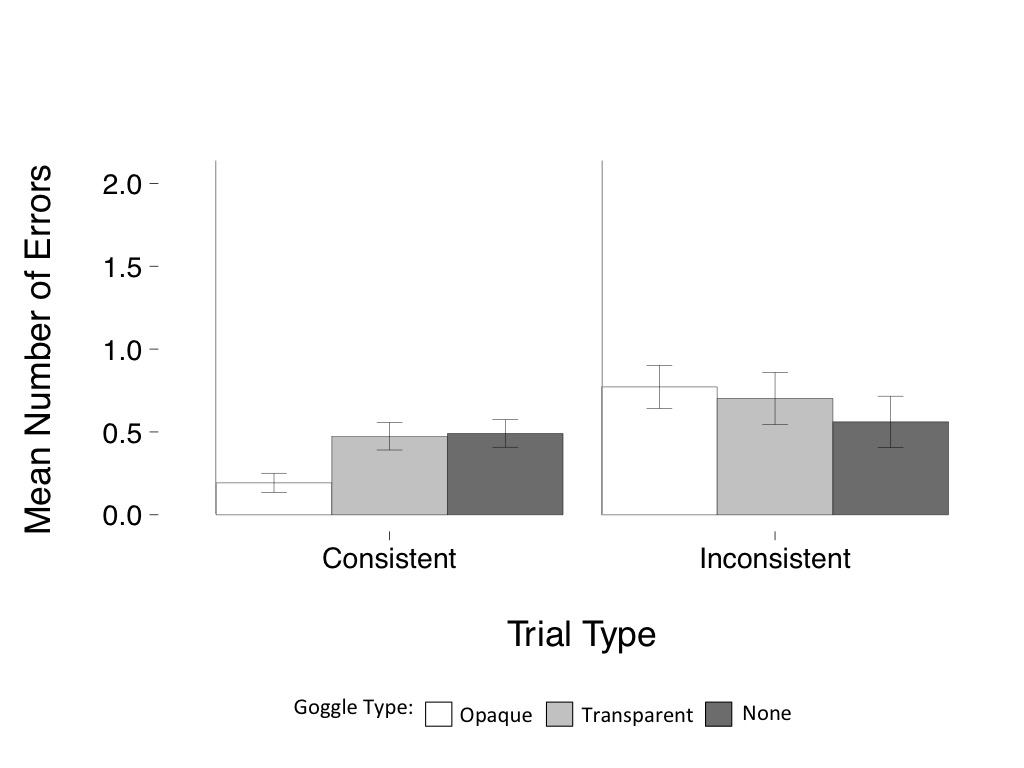
 Figure S.3.* Error bars show the Standard Error of the Mean.

**Table S.6.**

*Experiment* *3 Accuracy Results from Repeated Measures ANOVA with Factors Consistency, Perspective, and Goggle Type.*

|  | *F* | *p* | *η_ρ_^2^* | *BF01* |
| --- | --- | --- | --- | --- |
| Consistency | 21.61 | < .001 | .278 | 2.295 x 10^-8^ |
| Perspective | 24.26 | < .001 | .302 | 3.675 x 10^-6^ |
| Goggle Type | 7.10 | .001 | .112 | 0.052 |
| Consistency x Perspective | 0.17 | .682 | .003 | 8.629 |
| Consistency x Goggle Type | 13.40 | < .001 | .193 | 0.037 |
| Perspective x Goggle Type | 5.11 | .007 | .084 | 0.530 |
| Consistency x Perspective x Goggle Type | 14.14 | < .001 | .202 | 0.007 |

*Note.* Consistency x Goggle Type in the Self condition: *F* _(2,112)_ = 0.828, *p* = .440, η_ρ_^2^ = .015, BF01 = 10.545; and in the Other condition: Consistency X Goggle Type: *F* _(2,112)_ = 19.84, *p* < .001, η_ρ_^2^ = .262, BF01 = 4.621 x 10^-4^.

**Figure S.4.** **Experiment 3 Mean Number of Errors** **for Each Consistency, Perspective, and Goggle Type**


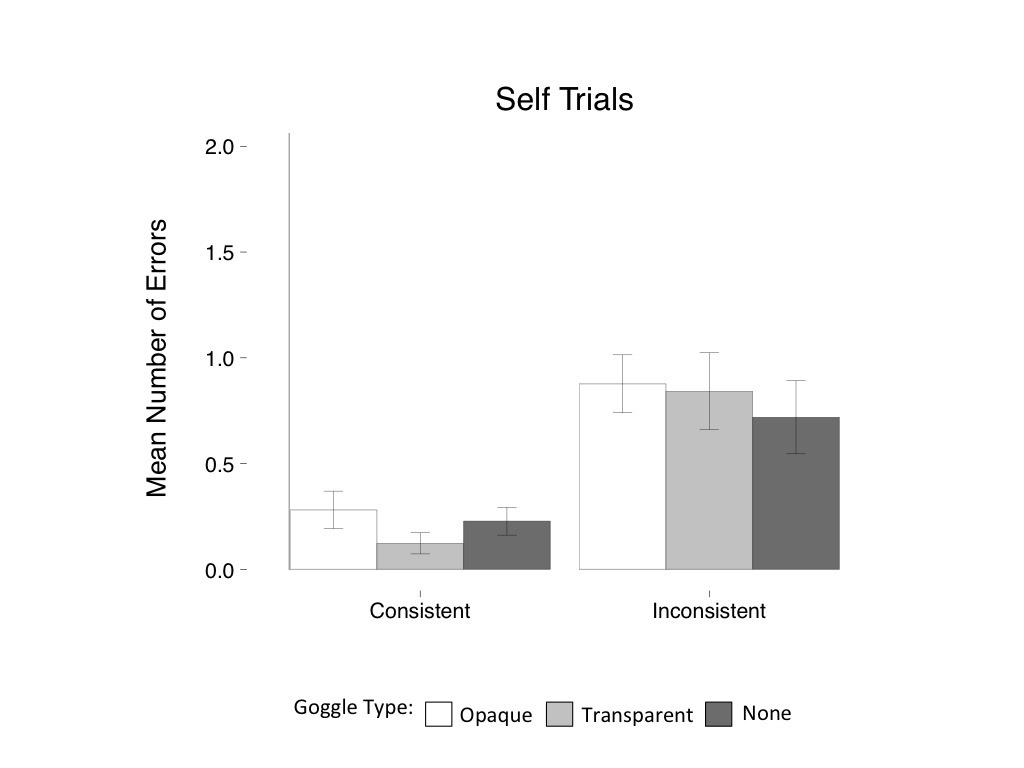


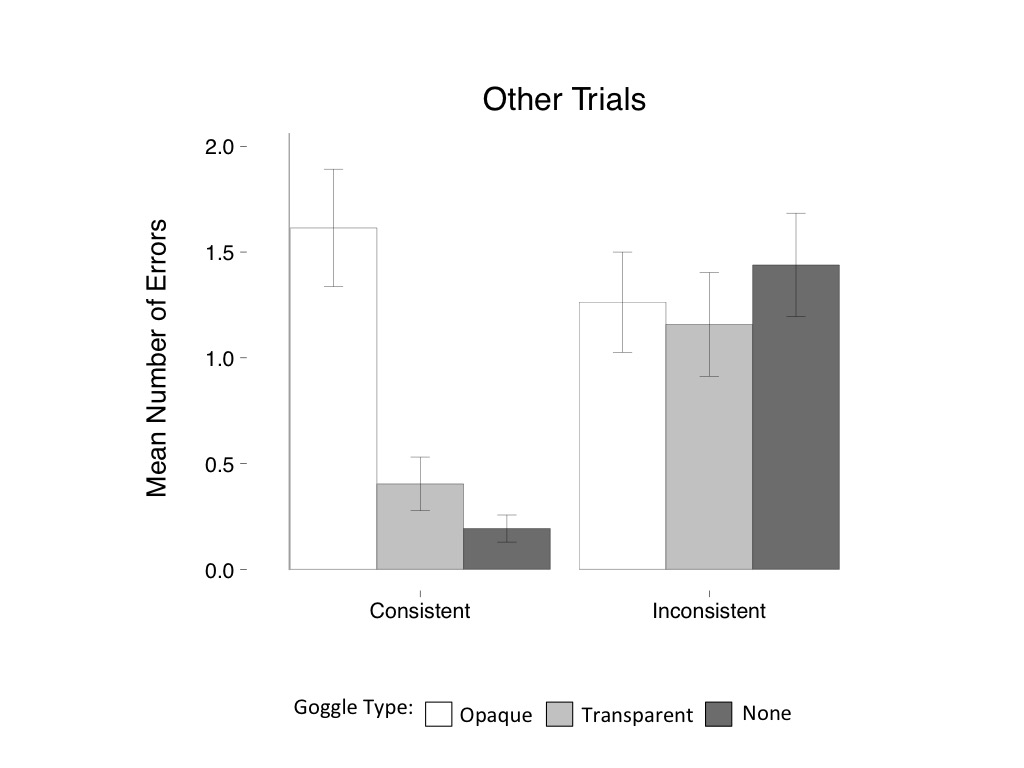


*Figure S.4.* Error bars show the Standard Error of the Mean.
